# Combination Therapy for Diabetic Macular Edema

**DOI:** 10.1155/2012/484612

**Published:** 2012-03-07

**Authors:** Dinah Zur, Anat Loewenstein

**Affiliations:** Department of Ophthalmology, Tel Aviv Medical Center, Sackler Faculty of Medicine, Tel Aviv University, 64239 Tel Aviv, Israel

## Abstract

Diabetic macular edema is a main reason for visual loss in diabetic patients. Until recent years, macular laser photocoagulation was the only available therapy. The awareness that inflammation is an important factor in the pathogenetic process of DME gave reason for intravitreal treatment with corticosteroids. The introduction of anti-VEGF drugs brought a revolutionary change in the treatment of DME. This paper will review the important clinical trials with an emphasis on combination therapies.

## 1. Introduction

Diabetic maculopathy is the main reason for visual loss in patients with diabetic retinopathy, besides proliferative diabetic retinopathy [1–3]. If left untreated, 25–30% of patients affected by diabetic macular edema (DME) experience a 15-letter decrease in visual acuity (VA) score within 3 years [4]. Strict control of glucose levels and blood pressure significantly reduces and delays the onset and severity of diabetic retinopathy [5, 6].

Available therapies include macular laser photocoagulation, corticosteroids, and anti-VEGF drugs. However, single treatments are often not effective enough to control DME during the entire course of the disease which can be very long. The multifactorial complex pathogenetic mechanisms require a comprehensive approach.

This paper will review the major trials and recent evidence evaluating both monotherapy and combination treatment for DME.

## 2. Available Monotherapies

### 2.1. Macular Laser Photocoagulation

Macular laser photocoagulation is a standard of care since shown in 1985 by the early treatment of diabetic retinopathy study (ETDRS) to reduce the risk of moderate visual loss in patients with clinically significant macular edema (CSME) by nearly 50% at 3 years. However, visual acuity (VA) improvement at 3 years, i.e., 15-letter gain, was found in less than 3% of cases [7]. This apparently slight improvement may be caused by the fact that 85% of patients had good entry vision (≥20/40). Still, 40% of those with entry VA ≥ 20/40 improved 1 or more lines. Yet, it should be considered that a notable number of patients stayed unresponsive to photocoagulation. As explicated below, the DRCR.net found focal/grid laser to be of greater benefit than monotherapy with triamcinolone [8]. With emergence of anti-VEGF treatment options the role of macular laser took a back seat in the treatment strategy.

### 2.2. Intravitreal Corticosteroids

The pathogenesis of DME is multifactorial. Breakdown of the blood-retina barrier increases retinal capillary permeability leading to retinal edema [9–11]. Inflammation is an eminent factor in this process, in particular via leukostasis within retinal capillaries [12]. The anti-inflammatory activity of corticosteroids is related to several paths of action: corticosteroids interfere with regulatory components of gene expression and inhibit the expression of proinflammatory genes as TNF*α* and other cytokines [13]. At the same time they induce gene functioning of anti-inflammatory factors, inhibit the phospholipase A_2_ pathway, and reduce leucocyte chemotaxis. Vitreous fluid levels of ICAM-1, IL-6, IL-8, and MCP-1 were found to be elevated in DME patients [14, 15]. On the other hand VEGF plays a major role in breakdown of the blood-retina barrier [16, 17]. Corticosteroids inhibit the expression of VEGF and VEGF gene [18, 19].

A multicenter randomized clinical trial by the Diabetic Retinopathy Clinical Research Network (DRCR.net) included 840 eyes and evaluated 1 mg and 4 mg doses of preservative-free triamcinolone compared with focal/grid photocoagulation for DME [20]. At four months, the 4 mg triamcinolone group had better visual acuity but by one year there were no significant differences. After 16 months and until the primary outcome visit at 2 years, mean visual acuity was better in the laser group than in the two triamcinolone groups. OCT results correlated with visual acuity. Recently, the DRCR.net published follow-up results of the third year. Findings were consistent with the 2-year results. More eyes in all groups improved than worsened. However, patients in the laser group had a benefit of +5 letters from baseline compared to the triamcinolone groups which stayed without change [8].

Expectedly, elevation of intraocular pressure and the need for cataract surgery were higher in the 4 mg triamcinolone group. These complications were likewise described in other studies [21, 22].

### 2.3. Intravitreal Anti-VEGF Treatment

A multitude of proinflammatory cytokines are involved in the development and progression of DME [23]. VEGF has been linked to leakage of retinal vessels and hence to the formation of retinal edema [24]. This was the rationale for testing anti-VEGF drugs for the treatment of DME.

#### 2.3.1. Ranibizumab

The *Safety and Efficacy of Ranibizumab in Diabetic Macular Edema* (RESOLVE Study)—multicenter, randomized, and double masked—evaluated the efficacy and safety of intravitreal ranibizumab (0.3 or 0.5 mg) compared with sham treatment (no ocular injection) in 151 eyes with DME over 12 months [25]. After three monthly injections treatment could be stopped or reinitiated with an opportunity for rescue macular laser photocoagulation. The dose could be doubled after one month. Results showed a significant and continuous improvement in BCVA and central retinal thickness for ranibizumab versus sham.

The phase III RIDE and RISE studies evaluated the efficacy and safety of ranibizumab for DME. RISE (*n* = 377) and RIDE (*n* = 382) are both double-blinded, sham-controlled randomized studies with a followup of 36 months. Patients received monthly injections of 0.3 mg ranibizumab, 0.5 mg ranibizumab, or sham. PRP was allowed when indicated, and rescue macular laser was permitted from month 3 onwards. 24-month results were recently presented [26]. In the RISE study, as twice as many patients in the ranibizumab groups gained ≥15 letters compared to the sham group (44.8%, 39.2%, and 18.1% in the ranibizumab 0.3 mg, 0.5 mg, and sham group, resp.). Results were similar in patients with better or less controlled glycemia. The RIDE study showed similar findings, but there was a clearer benefit for patients treated with 0.5 mg ranibizumab. Moreover, in the ranibizumab groups there were significantly more patients that achieved VA ≥ 20/40 compared to sham (60% and 63.2% versus 37.8% in RISE and 54.4% and 62.2% versus 34.6% in RIDE). Ranibizumab injections also reduced the percentage of patients progressing to proliferative diabetic retinopathy.

#### 2.3.2. Bevacizumab

The Pan-American Collaborative Retina Study Group (PACORES) reviewed 139 eyes with DME at 11 centers which received at least one bevacizumab injection of 1.25 or 2.5 mg with a minimum followup of 24 months [27]. Results showed that at 24 months 44.6% eyes remained stable, 51.8% improved 2 or more ETDRS lines, and 3.6% decreased 2 or more lines. Anatomic changes on OCT paralleled overall functional improvement.

The Bevacizumab or Laser Therapy in the Management of Diabetic Macular Edema study (BOLT study) is to date the most meaningful study concerning Bevacizumab for DME [28]. As a prospective and masked clinical trial with follow-up of 12 months it randomized 80 patients with CSME and at least one prior macular laser photocoagulation to Bevacizumab injections given every 6 weeks or laser treatment performed every 4 months. There was a clear benefit for the Bevacizumab group regarding BCVA improvement and CMT decrease versus the laser group.

## 3. Combination Therapy

### 3.1. Intravitreal Triamcinolone plus Macular Laser Photocoagulation

A randomized controlled trial evaluated the clinical outcome of macular laser photocoagulation after intravitreal triamcinolone acetonide (IVTA) for diffuse DME [29]. 86 eyes were included and randomized to two groups: the laser group patients underwent IVTA and macular grid photocoagulation 3 weeks afterward; the control group patients were treated with IVTA only. The interval of 3 weeks was chosen because of maximal therapeutic effects of IVTA at that time point.

Mean CMT was 538 *μ* at baseline, 250 *μ* after 3 weeks, 295 and 301 *μ* at 3 and 6 months after IVTA in the laser group versus 510, 227, 302, and 437 *μ* in the control group. Visual acuities were significantly better in the laser group after 3 and 6 months. Though followup was short, it seems that combination therapy maintains reduced CMT three months after IVTA injection. As expected, almost 40% of patients in both groups suffered IOP rise above 21 mmHg; one patient was referred to trabeculectomy.

### 3.2. Intravitreal Ranibizumab plus Macular Laser Photocoagulation (READ2)

The multicenter, prospective, randomized* Ranibizumab for Edema of the mAcula in Diabetes-2 (READ2) study *(phase II) compared the efficacy of intravitreal ranibizumab with focal/grid laser and a combination of both in 126 patients with DME [30]. Patients in the ranibizumab group received 0.5 mg ranibizumab at baseline and at months 1, 3, and 5. The laser group was treated with focal/grid laser photocoagulation at baseline and at month 3 if needed. The combination treatment consisted of focal/grid laser and ranibizumab injections at baseline and at month 3. After the primary endpoint at 6 months, all patients could receive ranibizumab injections according to retreatment criteria: patients in the ranibizumab group could have ranibizumab injections, patients in the laser group could have laser or ranibizumab, and patients in the combination group could have laser plus ranibizumab or ranibizumab alone. 

At month 6, the ranibizumab group gained significantly more BCVA than the laser and the combination group: 22% improved ≥3 lines compared to 0% and 8%, respectively. At two years, the visual outcome was not significantly different in the three groups; the percentages of patients who gained ≥3 lines rose to 24, 18, and 26. The mean improvement of BCVA was 7.4, 0.5, and 3.8 letters, respectively, at 6 months and 7.7, 5.1, and 6.8 letters at two years. 45%, 44%, and 35% reached a Snellen equivalent of 20/40 or better. Though there was a substantial decrease of mean foveal thickness in the ranibizumab group at 6-month, it increased during the further followup. In contrast, there was a constant decline in the two other groups during 24 months. This implicates that additional macular laser photocoagulation is auxiliary to decrease persistent or recurrent DME and to reduce the number of required injections. Furthermore the results show that patients with recurrent or persistent DME after 6 months ranibizumab treatment maintain their visual acuity gain by means of treatment continuation every two months during a period of 18 months. Patients treated previously by macular laser photocoagulation experienced a significant improvement of visual acuity after 18 months of ranibizumab treatment. 

### 3.3. Ranibizumab plus Prompt or Deferred Macular Laser Photocoagulation versus Triamcinolone plus Macular Laser Photocoagulation (DRCR.Net)

The DRCR.net conducted a multicenter, randomized clinical trial which included 854 eyes of 691 patients [31]. Eyes were randomized into four treatment groups: prompt laser with sham injection, 0.5 mg of ranibizumab with prompt laser, 0.5 mg of ranibizumab with laser deferred for at least 24 weeks, and 4 mg of triamcinolone with prompt laser. At one year, the two groups treated with ranibizumab had a significant change in mean VA from baseline. The triamcinolone and laser alone groups did not show a significant change in VA. Likewise, significantly more patients gained ≥10 letters in the two ranibizumab groups than in the laser alone group (50% and 47% versus 28%), and less patients lost ≥10 letters (4% and 3% versus 13%). Notably, a subgroup analysis of pseudophakic eyes in the triamcinolone group showed similar results as for those in the ranibizumab groups. OCT results paralleled visual acuity.

### 3.4. Ranibizumab plus Macular Laser Photocoagulation (RESTORE)

Similarly, the RESTORE study was a randomized, double-masked, multicenter phase III study over 12 months that compared ranibizumab + sham laser and ranibizumab + laser with laser + sham injection for DME in 345 patients [32]. Ranibizumab or sham injections were given monthly for three months and then PRN; laser or sham laser was given at baseline and then PRN after an interval of at least three months. In the ranibizumab and ranibizumab + laser groups a rapid improvement of VA was observed after one month which continued up to three months and was sustained until month 12 (6.8 ± 8.3 and 6.4 ± 11.8 letter gain, resp.), (Figure 3). The laser group maintained stable VA and gained 0.9 letters at month 12. Likewise, the percentage of patients reaching VA ≥ 20/40 was greater in the two ranibizumab groups (53% in the ranibizumab group and 44.9% in the ranibizumab + laser group versus 23.6% in the laser group). Accordingly, CMT decreased significantly in the two laser groups. This study evaluated also health-related quality of life by means of a questionnaire and found a greater improvement in the two ranibizumab groups. The number of PRN injections was about four in all three groups; the need for PRN laser was also similar in all groups. Summing up, ranibizumab monotherapy and combination with laser treatment are superior to laser treatment alone for DME. No differences in efficacy were found between the two ranibizumab groups.

### 3.5. PRP plus Macular Laser Photocoagulation plus Ranibizumab or Triamcinolone

**Figure 1:**
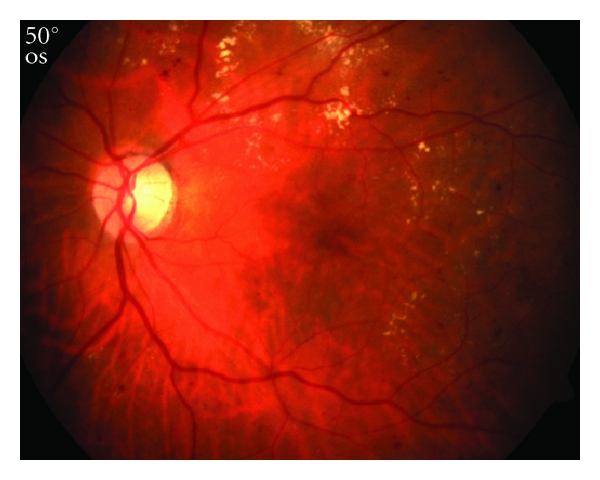
Fundus photograph of the left eye from a patient with severe NPDR and CSME.

The DRCR.net recently published short-term results (14 weeks) of a phase 3, randomized, multicenter, clinical trial which addressed DME in conjunction with panretinal photocoagulation (PRP) [33]. The study included 340 eyes with CSME and severe NPDR or PDR (Figure 1). Patients were randomized to receive sham injections or 0.5 mg ranibizumab at baseline and at 4 weeks or 4 mg IVTA at baseline and sham injection at 4 weeks. Macular laser was performed within 3 to 10 days, and PRP was initiated immediately or within 14 days of the baseline injection. Mean change in visual acuity letter score from baseline was significantly better in the ranibizumab and IVTA groups. These two groups also had a greater proportion of eyes which improved ≥10 letters and a lower proportion of eyes which worsened ≥10 letters at 14 weeks. CMT changes behaved similarly. After 14 weeks of followup patients were evaluated after 56 weeks for safety information. At this point, differences were not maintained.

### 3.6. Intravitreal Bevacizumab plus Triamcinolone

A clinical trial by Soheilian et al. randomized 150 eyes with naïve CSME into three groups: Bevacizumab + sham laser, bevacizumab + IVTA + sham laser, and macular laser photocoagulation + sham injection [34]. Retreatment was performed at 12 weeks as needed. Change in BCVA at 24 weeks as the primary outcome measure showed a significant improvement in the bevacizumab groups at all follow-up visits up to 36 weeks. There was no significant difference between the bevacizumab and the combination group. The laser group did not have a significant VA change. A statistically significant greater percentage of patients gained ≥2 lines in the bevacizumab groups whereas a greater percentage of patients lost ≥2 lines in the laser group. All groups experienced a significant decrease of CMT at 6 weeks-without a significant difference between the groups.

Based upon the finding that visual outcome at 24 months was better for patients treated with bevacizumab (alone or in combination with IVTA) than with laser, the authors recommend considering bevacizumab as a first-line treatment. The addition of IVTA did not have an adjunctive effect. However, it should be noted that the study did not assess combination of macular laser with bevacizumab or IVTA as done by the DRCR.net. Another limiting factor is the relative short followup.

### 3.7. Bevacizumab plus Macular Laser Photocoagulation

**Figure 2:**
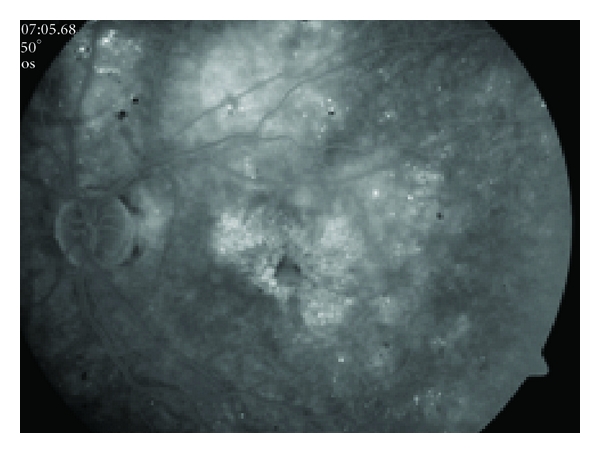
Late phase of fluorescence angiography shows macular leakage with a petalloid pattern. Note multiple microaneurysms and diffuse hyperfluorescence superotemporal to optic disc.

**Figure 3:**
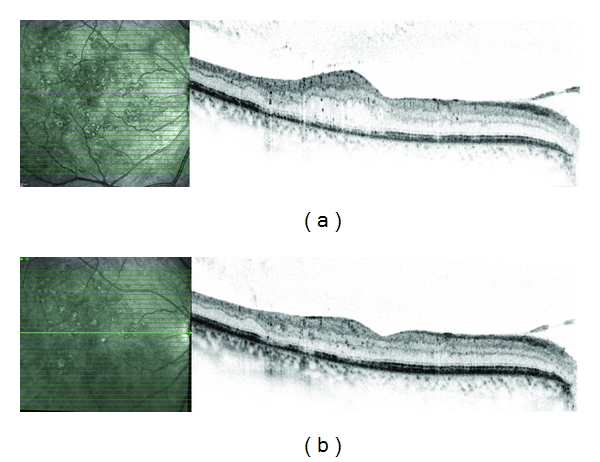
High-resolution OCT of the right eye of a patient with CSME. (a) shows loss of foveal contour and cystoid edema. (b) One month after intravitreal avastin injection foveal contour is restored, retinal thickening decreased significantly, and there are no cystoids spaces detected anymore.

A smaller clinical trial including 62 eyes with diffuse DME evaluated the efficacy of combined bevacizumab plus macular laser photocoagulation versus each alone as primary treatment [35]. At month 1, patients in the bevacizumab group and in the combined group experienced significant reduction in CMT—in contrast to patients treated by laser only. Accordingly, the two groups treated with bevacizumab had significant improvement of VA whereas the laser group did not have significant change. After three and six months, decrease of CMT was significant only in the combined group. A trend towards advantage for combined treatment was also noticed with VA improvement and decrease of macular leakage on FA (Figure 2). At six months, VA even regressed to baseline values in the laser and bevacizumab groups whereas the improvement in the combined group was not statistically significant. It should be noted that there was no masking by sham treatment in this study.

## 4. Discussion

Since the results of the ETDRS, macular laser photocoagulation has been considered the mainstay of treatment for DME although visual outcomes have not been satisfactory [4]. The introduction of intravitreal corticosteroids and anti-VEGF treatments changed the perspective on macular edema in general and on DME in particular.

Though the studies mentioned above had different designs, some findings are consistent.

Ranibizumab monotherapy and ranibizumab in combination with laser are more effective than macular laser photocoagulation monotherapy. For bevacizumab, superiority has been shown for combination with macular laser photocoagulation to each of them alone.

The additive synergistic effect of laser to anti-VEGF treatment can be explained by several mechanisms. Decreased foveal thickness facilitates laser treatment and reduces the need for high laser energy. Furthermore, more marked and prolonged reduction in macular thickness is achieved. Bevacizumab and ranibizumab downregulate VEGF and reduce capillary permeability. However, VEGF is only one factor in a complex pathogenetic process, and macular hypoxia as an underlying problem is not addressed. This may explain the rapid recurrence of macular edema a few weeks after injection when VEGF levels again increase in the vitreous. Grid laser photocoagulation decreases oxygen consumption by destroying photoreceptors. Hence, combining anti-VEGF with laser photocoagulation is a complementary treatment with high efficacy in treating DME and decreasing recurrence.

Combination therapy of IVTA plus macular laser is more effective than either monotherapy and may be comparable to anti-VEGF plus laser in pseudophakic patients. The success of this combination therapy may be due to several mechanisms: IVTA decreases foveal thickness and allows more precise and effective macular laser photocoagulation with lower energy levels needed. Furthermore, steroids might promote the formation of mature laser scars. A deterioration of macular edema is a well-known complication of laser treatment. Intravitreal therapeutic levels of steroids seem to be protective and even modulate the RPE modeling after laser.

PRP frequently causes exacerbation of DME. Prompt laser with IVTA or ranibizumab treatment results in better visual acuity and reduced CMT in the short term.

Though intravitreal corticosteroids and anti-VEGF drugs have different ways of action there was no adjunctive effect found with combination therapy.

Since macular laser photocoagulation was first established by the ETDRS, treatment strategy for DME has essentially changed. Different treatment approaches reflect the complex pathogenesis of the disease. However, refractory cases still exist even with combination therapies. Due to heterogeneity among patients there will be no ideal treatment regimen for everybody. Further investigation of additional treatment options is needed in order to optimize therapy.
